# Abnormal Expression of YAP Is Associated With Proliferation, Differentiation, Neutrophil Infiltration, and Adverse Outcome in Patients With Nasal Inverted Papilloma

**DOI:** 10.3389/fcell.2021.625251

**Published:** 2021-04-15

**Authors:** Tian Yuan, Rui Zheng, Xiang-min Zhou, Peng Jin, Zhi-qun Huang, Xiao-xue Zi, Qing-wu Wu, Wei-hao Wang, Hui-yi Deng, Wei-feng Kong, Hui-jun Qiu, Sui-zi Zhou, Qian-min Chen, Yan-yi Tu, Tao Li, Jing Liu, Kai Sen Tan, Hsiao Hui Ong, Li Shi, Zhuang-gui Chen, Xue-kun Huang, Qin-tai Yang, De-yun Wang

**Affiliations:** ^1^Department of Otolaryngology-Head and Neck Surgery, Department of Allergy, The Third Affiliated Hospital of Sun Yat-sen University, Guangzhou, China; ^2^Department of Otolaryngology, Yong Loo Lin School of Medicine, National University of Singapore, Singapore, Singapore; ^3^Department of Otolaryngology-Head and Neck Surgery, Shandong Provincial ENT Hospital, Cheeloo College of Medicine, Shandong University, Jinan, China; ^4^Department of Otolaryngology-Head and Neck Surgery, The First Affiliated Hospital of Nanchang University, Nanchang, China; ^5^Department of Otolaryngology, Zhujiang Hospital, Southern Medical University, Guangzhou, China; ^6^NUHS Infectious Diseases Translational Research Program, Yong Loo Lin School of Medicine, National University of Singapore, Singapore, Singapore; ^7^Department of Microbiology and Immunology, Yong Loo Lin School of Medicine, National University of Singapore, Singapore, Singapore; ^8^Biosafety Level 3 Core Facility, Yong Loo Lin School of Medicine, National University Health System, National University of Singapore, Singapore, Singapore; ^9^Department of Pediatrics, Department of Allergy, The Third Affiliated Hospital of Sun Yat-sen University, Guangzhou, China

**Keywords:** nasal inverted papilloma, yes-associated protein, epithelial cells, proliferation, differentiation, neutrophils

## Abstract

**Background:**

Nasal inverted papilloma (NIP) is a common benign tumor. Yes-associated protein (YAP) is the core effector molecule of the Hippo pathway, which regulates the proliferation and differentiation of airway epithelium. While its role in proliferation may be connected to NIP formation, no definitive association has been made between them.

**Methods:**

We compared the difference of YAP expression and proliferation level between the control inferior turbinate, NP (nasal polyps), and NIP groups. In addition, we further used PCR, immunofluorescence, and immunohistochemistry to investigate YAP’s role in the proliferation and differentiation of the nasal epithelium and inflammatory cell infiltration, correlating them with different grades of epithelial remodeling. We further used an IL-13 remodeling condition to investigate YAP’s role in differentiation in an *in vitro* air-liquid interface (ALI) human nasal epithelial cell (hNECs) model. Finally, we also explored the correlation between YAP expression and clinical indicators of NIP.

**Results:**

The expression of YAP/active YAP in the NIP group was significantly higher than that in the NP group and control group. Moreover, within the NIP group, the higher grade of epithelial remodeling was associated with higher YAP induced proliferation, leading to reduced ciliated cells and goblet cells. The finding was further verified using an IL-13 remodeling condition in differentiating ALI hNECs. Furthermore, YAP expression was positively correlated with proliferation and neutrophil infiltration in NIP. YAP expression was also significantly increased in NIP patients with adverse outcomes.

**Conclusion:**

Abnormal expression of YAP/active YAP is associated with proliferation, differentiation, neutrophil infiltration, and adverse outcome in NIP and may present a novel target for diagnosis and intervention in NIP.

## Introduction

Nasal inverted papilloma (NIP) is a benign epithelial tumor growing in the nasal cavity and sinuses, characterized by their local invasiveness, recurrence, and malignant transformation (Katori et al., 2006; Sun et al., 2017). NIP grows as an extraneous polypoid with an endophytic (inverted) growth pattern. NIP can be formed from epithelial cells of different tissue origins, including squamous epithelium, respiratory epithelium, and transitional epithelium (Nielsen et al., 1991). At present, the etiology of NIP is unclear. Some researchers hold the view that NIP is a kind of tumor-derived from the Schneider’s membrane; some think that NIP may be the result of an initial inflammatory response (Orlandi et al., 2002). Additionally, it was also found that HPV may be responsible for causing NIP’s malignant transformation (Zhao R. W. et al., 2016). While NIP can be surgically treated, the disease is often easily misdiagnosed as a nasal polyp because of their similar clinical features (Paz Silva et al., 2015). However, compared with nasal polyps, NIP has a higher recurrence and malignancy rate. Many studies have found that the proliferation level of NIP is significantly higher than that of nasal polyps, which may be an important reason for the recurrence and malignant transformation of NIP (Mumbuc et al., 2007; Meng et al., 2014). Our previous research showed that inflammatory cells were identified as a significant cell population in NIP (Zhao L. et al., 2016), which supports the hypothesis that NIP formation is associated with inflammatory response (Orlandi et al., 2002). However, the mechanism of the high proliferation level and abnormal inflammatory cell infiltration in NIP is still unclear.

Yes-associated protein (YAP) is a transcriptional co-activator in the conserved Hippo pathway, which was shown to regulate cell proliferation, cell differentiation, and apoptosis (Yu et al., 2015). In airway epithelial stem cells, the Hippo pathway plays an important role in the self-renewal of stem cells and progenitor cells, as well as maintaining the balance between undifferentiated and differentiated cells. During the development of mouse lower airway, YAP can control the fate of lower airway epithelial progenitor cells and airway morphogenesis (Mahoney et al., 2014). In the mature lower airway of mice, YAP deletion leads to the loss of basal stem cells and uncontrolled differentiation. On the contrary, the overexpression of YAP strengthens the self-renewal and inhibits the differentiation of basal stem cells, resulting in epithelial proliferation and the formation of multi-layer undifferentiated cells (Zhao R. et al., 2014; Lange et al., 2015). In the nasal epithelium (upper airway), our team found that the expression of YAP in nasal polyps was abnormal and was involved in the epithelial proliferation and tissue remodeling of nasal polyps (Deng et al., 2019). Furthermore, it was also found that the Hippo pathway plays a role in inflammation. For example, defects in MST1 (mammalian sterile 20-like kinase 1), an upstream factor of YAP, leads to the downregulation of neutrophils (Kurz et al., 2018), and the specific knockout of YAP in vascular endothelium will lead to an increase of neutrophil infiltration (Lv et al., 2018). Thus, YAP has been shown to affect the airway proliferation and inflammation, which may likely be associated with NIP pathogenesis. Therefore, the present study aims to investigate the relationship between YAP and proliferation, differentiation, and inflammation in NIP pathogenesis.

## Materials and Methods

### Patients and Samples

Control subjects (inferior turbinate, IT), patients with nasal polyps (NP), and patients with NIP were recruited from the Qilu Hospital of Shandong University (Jinan, China) and 3rd Affiliated Hospital of Sun Yat-sen University (Guangzhou, China). Control samples were obtained from healthy inferior turbinate tissues of patients who underwent septal plastic surgery. The diagnosis of NP and NIP was confirmed and reported by a pathologist. Other types of nasal papilloma (exophytic and cylindrical cell papilloma) were excluded from the study. The nasal polyps group had no concurrent NIP. The samples of NP and NIP were from different individuals. For patients with recurrent NIP, they went through NIP surgery before specimen collection. All patients had not used any form of glucocorticosteroids or antibiotics within 3 months prior to specimen collection.

The NIP clinical stages I to IV was evaluated according to previous studies (Krouse, 2000). In addition, we further based the evaluation on the results of patients’ computed tomography (CT) scans and endoscopic examination. History of recurrence was obtained with patient records and confirmed by outpatient and intraoperative endoscopic evidence of prior nasal surgery. Smokers were defined as current cigarette smokers who consume one or more packs of cigarettes a day, averaged over 1 year. Fresh specimens were fixed in formalin and preserved in RNAlater solution for histologic evaluation and detecting gene expression, respectively. Approval for this study was obtained from the institutional review boards of Qilu Hospital of Shandong University (2019124 Jinan, China) and 3rd Affiliated Hospital of Sun Yat-sen University ([2016]2–40, Guangzhou, China). Each subject provided written informed consent before participation.

### Remodeling Evaluation of NIP Epithelium

The NIP was graded into three categories based on comprehensive results of hematoxylin and eosin (HE)-staining and immunofluorescent staining, according to a previous study (Roh et al., 2004; Figures 2A,E). Grade I was defined as ciliated respiratory epithelium with underlying squamous metaplasia; Grade II as partially ciliated respiratory epithelium with luminal squamous metaplasia and increased prominence of inversion; and Grade III as almost complete absence of respiratory epithelium with dominant stratified squamous epithelium. For each NIP specimen, the grading of remodeling was based on the predominant remodeling present (>70% of the epithelium showing this feature).

### RNA Extraction and Quantitative Real-Time Polymerase Chain Reaction

Total RNA was extracted from frozen nasal tissues in RNA later. Then, 1,000 ng of total RNA was reverse transcribed into cDNA using Maxima Reverse Transcriptase Kit (Thermo Fisher Scientific) according to manufacturer’s protocol. The mRNA level was detected by SYBR green gene expression assays. Relative gene expression was calculated using the comparative 2^–ΔΔ^ ^Ct^ method normalized against the housekeeping gene [ribosomal protein L13a (RPL13A)]. The primers sequences were as follows: YAP forward (5′-AATTGAGAACAATGACGACC-3′), YAP reverse (5′-AGTATCACCTGTATCCATCTC-3′); Ki-67 forward (5′-ACGAGACGCCTGGTTACTATC-3′), Ki-67 reverse (5′-GCTCATCAATAACAGACCCATTTAC-3′); RPL13A forward (5′-GTCTGAAGCCTACAAGAAAG-3′), RPL13A reverse (5′-TGTCAATTTTCTTCTCCACG-3′).

### Human Nasal Epithelial Cells Culture and IL-13 Treatment

Human nasal epithelial cells (hNECs) were developed from primary human nasal epithelial stem/progenitor cells (hNESPCs) (Li et al., 2014; Liu et al., 2018; Yuan et al., 2020), obtained from fresh healthy inferior turbinate mucosa. The hNESPCs were transplanted to an air-liquid interface (ALI) system to form a pseudostratified layer within 4 weeks. A detailed description of the hNEC culture method used is found in our previously published papers (Li et al., 2014; Liu et al., 2018; Yuan et al., 2020). To establish a remodeling condition *in vitro*, IL-13 (10 ng/mL, R&D System, Minneapolis, MN, United States) was added to the basal cell culture medium of the hNECs during their entire duration of differentiation.

### Immunofluorescence Staining and Analysis

Protein expression of YAP (sc101199, Santa Cruz Biotechnology), active YAP (ab205270, Abcam), P63 (ab124762, Abcam), Ki-67 (ab15580, Abcam), β4-TUBULIN (ab179504, Abcam), and MUC5AC (sc20118, Santa Cruz Biotechnology) on paraffin tissue sections were examined by immunofluorescence staining (IF). All the sections were blocked using 10% normal goat serum for 30 min at room temperature. They were then incubated with a primary antibody solution overnight at 4°C, followed by 1 h incubation with Alexa Fluor 488- or 594-conjugated secondary antibodies in the dark at room temperature. Cellular nuclei were visualized using 4′,6-diamidino-2-phenylindole (DAPI) (Life Technologies, Carlsbad, CA, United States). For negative controls, primary antibodies were substituted with the species- and subtype-matched antibodies at the same concentration. The slides were analyzed with fluorescent microscopy (Olympus IX51, Tokyo, Japan).

Images of YAP on tissue sections were captured at ×400 magnification with a fluorescence microscope (Olympus IX51, Tokyo, Japan). Fluorescence intensity was performed by YAP antibody (sc101199, Santa Cruz Biotechnology) and was analyzed using ImageJ software (National Institutes of Health, Bethesda, MD, United States) through calculating the raw mean fluorescence intensity (rMFI) in YAP IF staining and the mean autofluorescence intensity (MAI) in negative controls. YAP expression was measured by corrected mean fluorescence intensity (MFI), which was equal to rMFI minus MAI. Percentage of nuclear active YAP was performed using active YAP antibody (ab205270, Abcam). Nuclear active YAP (nuclear-aYAP) was only counted in the epithelium. For every sample, MFI and nuclear-aYAP were averaged from three images. Clinical characteristics of patients used in YAP MFI and qPCR were listed in Table 1. Patient characteristics used in calculating the percentage of nuclear active YAP were listed in Supplementary Table 1.

**TABLE 1:** Clinical characteristics of patients.

Clinical parameters	Control (*n* = 10) No. (%)	NP (*n* = 20) No. (%)	NIP (*n* = 29) No. (%)
Age, year, median (1st and 3rd interquartile)	35 (20,50)	42 (29,52)	47 (39,55)
**Gender**			
Male	8 (80%)	15 (75%)	23 (79%)
Female	2 (20%)	5 (25%)	6 (21%)
**Smoking**			
Smoker	2 (20%)	4 (20%)	11 (38%)
Non-smoker	8 (80%)	16 (80%)	18 (62%)
**Concurrent CRS**			
Yes	–	–	9 (31%)
No	–	–	20 (69%)
**Concurrent NP**			
Yes	–	–	8 (28%)
No	–	–	21 (72%)
**Krouse staging system**			
Stage I	–	–	1 (3%)
Stage II	–	–	1 (3%)
Stage III	–	–	24 (83%)
Stage IV	–	–	3 (10%)

*NIP, nasal inverted papilloma; CRS, chronic rhinosinusitis; NP, nasal polyps.*

### Immunohistochemistry Staining and Evaluation of Inflammatory Cells

Evaluation of inflammatory cells was examined by immunohistochemistry staining (IHC), except eosinophils were measured by HE–staining. The specific inflammatory cell markers were as follows: mouse monoclonal anti-human neutrophil elastase (clone NP57) (Dako, Glostrup, Denmark) for neutrophils, mouse monoclonal anti-human CD68 (clone KP1) (Abcam, Cambridge, United Kingdom) for macrophages, mouse monoclonal anti-human CD4 (clone 4B12) (Dako) for helper T cells, mouse monoclonal anti-human CD8 (clone C8/144B) (Thermo Scientific, Fremont, CA, United States) for cytotoxic T cells, and mouse monoclonal anti-human Foxp3 [clone, 236A/E7] (Abcam) for regulatory T cells.

Nasal inverted papilloma tissue was embedded in paraffin and sectioned at 4 μm with a Leica microtome (Leica, Wetzlar, Germany). Paraffin tissue sections were blocked using 10% normal goat serum for 30 min at room temperature. They were pretreated with Target Retrieval Buffer (Dako) and then incubated with a primary antibody solution overnight at 4°C. The next day the cellular markers were stained by using a modified horseradish peroxidase (HRP) technique with the DakoCytomation EnVision1System-HRP (Dako). Species- and subtype-matched antibodies were used as negative controls (Dako). The slides were then incubated with Dako EnVision + System-HRP (Dako) at room temperature for 30 min, after which the substrate diaminobenzidine was added for color development. All slides were counterstained with hematoxylin.

When evaluating inflammatory cells, we first used low magnification to locate the areas with the most severe infiltration of inflammatory cells. Then observed 3 high-power fields and counted at least 300 leukocytes. Epithelial cells, fibroblasts, glandular cells, and endothelial cells were excluded. Of the 3 fields, the proportion of each type of inflammatory cell is the ratio of all this type of cell to all leukocytes. For IF and IHC, two researchers evaluated the data independently, and the third researcher independently evaluated and solved any disagreement between the first two researchers.

### Statistical Analysis

All data were analyzed using GraphPad Prism 8 (GraphPad Software, La Jolla, CA, United States). For comparison of differences between two groups, Mann–Whitney test and Wilcoxon matched-pairs signed rank test were applied. Kruskal–Wallis test was used to analyze differences between multiple groups. Correlation analysis was performed using the Spearman *r* test. A *P-*value of <0.05 was considered statistically significant.

## Results

### YAP Is Overexpressed in Nasal Inverted Papilloma

**FIGURE 1:**
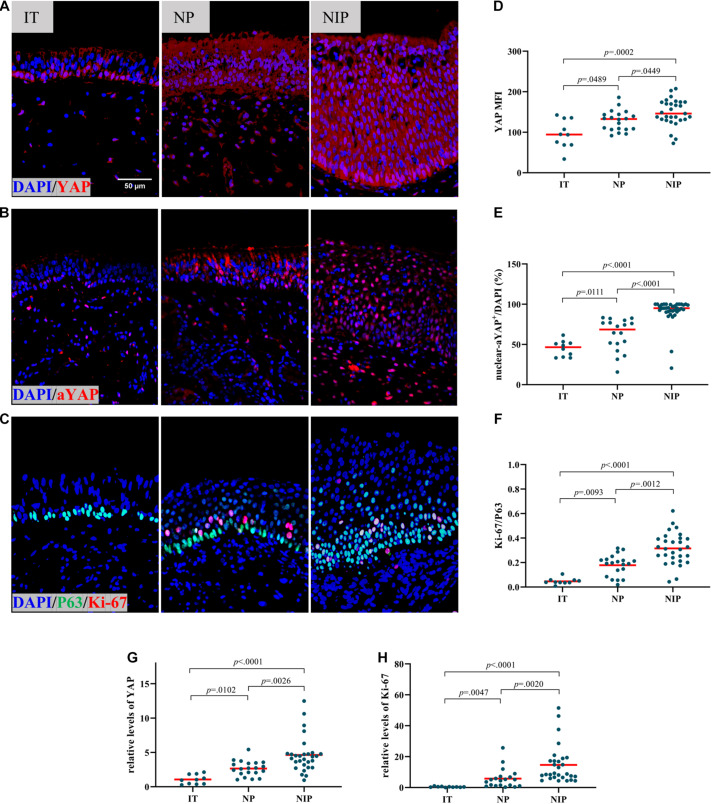
YAP is overexpressed in nasal inverted papilloma. **(A,B)** Total YAP and active YAP IF staining in control IT, NP, and NIP tissues. **(C)** Ki-67 and P63 double IF staining in control IT, NP, and NIP tissues. **(D–F)** Semi-quantitative analysis of mean fluorescence intensity (MFI) stained for total YAP, percentage of nuclear active YAP positive cells (nuclear-aYAP^+^/DAPI), ratio of Ki-67^+^ cells to P63^+^ cells (Ki-67/P63) on IF staining in control IT, NP, and NIP tissues. **(G,H)** The mRNA levels of YAP and Ki-67 in control IT, NP, and NIP tissues were quantified by RT-qPCR assays and relative expression of the target gene was normalized to 2^− Δ^ ^CT^ with RPL13A. Multiple group comparison was using Kruskal–Wallis test. Red lines show median values. **(A–C)**, ×400 magnification, scale bar = 50 μm. **(D,F–H)**, *n*(IT) = 10, *n*(NP) = 20, *n*(NIP) = 29. **(E)**, *n*(IT) = 10, *n*(NP) = 18, *n*(NIP) = 36.

We first evaluated YAP expression in control inferior turbinate (IT), nasal polyps (NP), and nasal inverted papilloma (NIP). In immunofluorescence staining (IF), epithelial MFI of YAP (Figures 1A,D) and percentage of nuclear active YAP positive cells (nuclear-aYAP^+^/DAPI) (Figures 1B,E and Supplementary Figure 1) in NIP was found to be higher than in IT and NP tissues. The same results could be observed at the mRNA level where YAP mRNA was increased in NIP compared to IT and NP (Figure 1G). Secondly, we examined the proliferation level of IT, NP, and NIP. We choose Ki-67, a widely recognized proliferation marker, co-stained with P63 to represent basal cells with proliferative ability. Thus, the ratio of Ki-67^+^ cells to P63^+^ cells (Ki-67/P63) would indicate the level of proliferation in the basal epithelium. Ki-67/P63 was increased in NIP compared with IT and NP (Figures 1C,F and Supplementary Figure 2), Ki-67/P63 in NP was also found to be higher than that in IT, congruent with our previous findings (Deng et al., 2019). The result of immunofluorescence staining was consistent with the PCR results. At the mRNA level, both NIP and NP showed increased Ki-67 expression, with NIP being higher than that of NP (Figure 1H). This trend of Ki-67 levels was consistent with YAP expression and indicated the correlation of high proliferation levels in NIP with YAP expression.

### Higher YAP Expression Level Is Associated With Reduced Nasal Epithelial Differentiation in NIP

**FIGURE 2:**
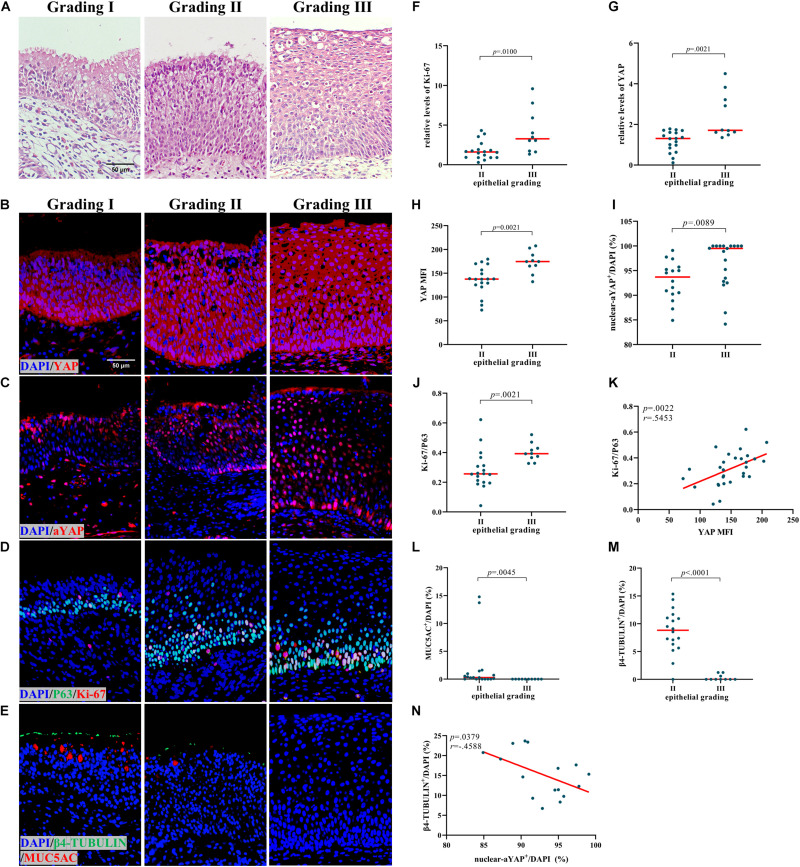
Increased YAP levels are associated with proliferation and differentiation in NIP. **(A)** HE staining in different epithelial remodeling grade of NIP tissues. **(B,C)** Total YAP and active YAP IF staining in different epithelial remodeling grade of NIP tissues. **(D,E)** Ki-67 and P63, β4-TUBULIN, and MUC5AC double IF staining in different epithelial remodeling grade of NIP tissues. **(F,G)** The mRNA levels of YAP and Ki-67 in control IT, NP, and NIP tissues were quantified by RT-qPCR assays and relative expression of the target gene was normalized to 2^− Δ^ ^CT^ with RPL13A. **(H–J,L,M)** Semi-quantitative analysis of mean fluorescence intensity (MFI) stained for total YAP, percentage of nuclear active YAP positive cells (nuclear-aYAP^+^/DAPI), ratio of Ki-67^+^ cells to P63^+^ cells (Ki-67/P63), goblet cell ratio (MUC5AC^+^/DAPI), ciliated cell ratio (β4-TUBULIN^+^/DAPI) on IF staining in Grade II and Grade III of NIP tissues. **(K)** Correlation analysis between YAP MFI and Ki-67/P63 on IF staining in NIP tissues. **(N)** Correlation analysis between nuclear-aYAP^+^/DAPI and β4-TUBULIN^ +^/DAPI on IF staining in Grade II NIP tissues. Two group difference was analyzed with Mann–Whitney test, and correlation analysis was performed using the Spearman *r* test. Red lines show median values. **(A–E)**, ×400 magnification, scale bar = 50 μm. **(F–H,J,L,M)**, *n*(Grade II) = 18, *n*(Grade III) = 10; **(I)**, *n*(Grade II) = 16, *n*(Grade III) = 19; **(K)**, *n* = 29; N, *n* = 16.

Based on a previous study, NIP can be further divided into three categories (Roh et al., 2004). The nasal epithelial remodeling was found to increase in severity from Grade I to Grade III accompanied by reduced nasal epithelial differentiation, and the representative HE and IF were shown in Figures 2A,E. The number and proportion of three types of NIP in this study were shown in Table 2 and Supplementary Table 1. As there were not enough Grade I samples, we only compared Grade II to Grade III. In Grade III NIP, YAP mRNA level (Figure 2F), YAP MFI (Figures 2B,H), and percentage of nuclear active YAP positive cells (nuclear-aYAP^+^/DAPI) (Figures 2C,I) were all significantly higher than that in Grade II.

**TABLE 2:** Clinical characteristics of patients with NIP.

Epithelial remodeling grading	No. (%) (*n* = 29)
Grade I	1 (3%)
Grade II	18 (62%)
Grade III	10 (34%)

In terms of proliferation, Grade III NIP has a higher level of Ki-67 than Grade II on both protein level (Figures 2D,J) and mRNA level (Figure 2G). We explored the relationship between YAP and proliferation in NIP. YAP MFI is positively correlated to Ki-67/P63 (Figure 2K). In terms of differentiation, ciliated cell ratio (β4-TUBULIN^+^/DAPI) (Figures 2E,M) and goblet cell ratio (MUC5AC^+^/DAPI) (Figures 2E,L) was decreased in Grade III compared with Grade II. While total YAP MFI was positively correlated with ciliated cell ratio in Grade II NIP (Supplementary Figure 3A), nuclear active YAP was found to be negatively correlated with ciliated cells ratio (Figure 2N). There was no significant difference between the goblet cell ratio and YAP MFI (Supplementary Figure 3B). For the goblet cell ratio, as the numbers are low in NIP groups, we did not explore its correlation with nuclear active YAP positive cells ratio. These results indicated that YAP may be involved in the severity of NIP via dysregulation of proliferation and differentiation of the nasal epithelium.

### Active YAP Is Negatively Correlated With Ciliated Cells in hNESPCs Culture Model

**FIGURE 3:**
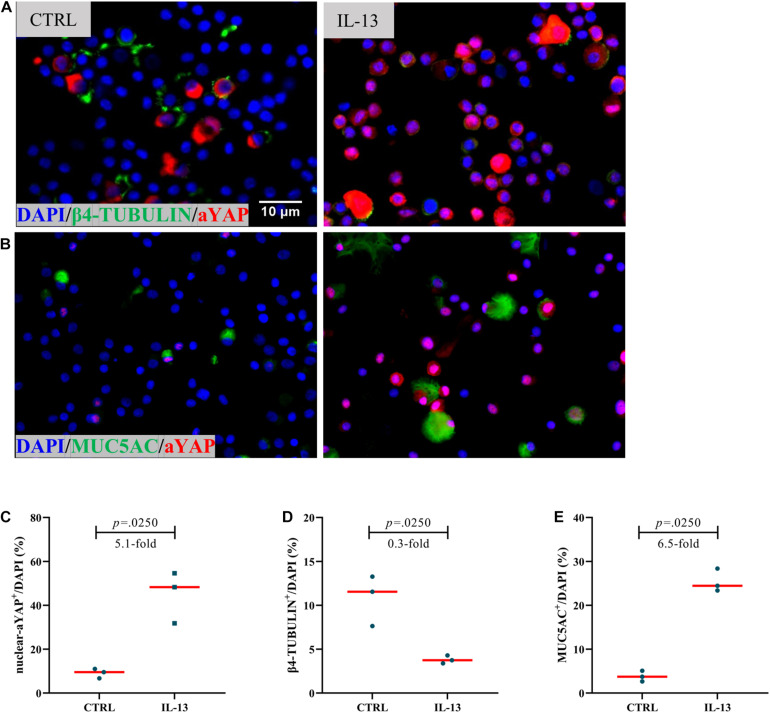
Increased active YAP is associated with differentiation in hNESPCs culture model. **(A,B)** β4-TUBULIN and active YAP (aYAP), MUC5AC, and aYAP double IF staining in control and IL-13 treatment group. **(C–E)** Percentage of nuclear active YAP positive cells (nuclear-aYAP^+^/DAPI), ciliated cell ratio (β4-TUBULIN^+^/DAPI), goblet cell ratio (MUC5AC^+^/DAPI) on IF staining in control and IL-13 treatment group. Two group difference was analyzed with Wilcoxon matched-pairs signed rank test, and fold-change was shown. Red lines show median values. **(A,B)**, ×800 magnification, scale bar = 10 μm. *n*(CTRL) = 3, *n*(IL-13) = 3.

A recent study has demonstrated that YAP/TAZ activity may be involved in the regulation of ciliogenesis (Kim et al., 2015). In order to explore the role of YAP during tissue remodeling processes in NIP, we used IL-13 on hNECs ALI cell culture. IL-13–mediated cellular remodeling of the human airway epithelium has been examined in multiple studies. IL-13 stimulation could induce more MUC5AC-positive mucus cells and fewer ciliated cells during the process of airway epithelia cell differentiation (Atherton et al., 2003; Dellagrammaticas et al., 2008). After the maturation of the hNECs, we found that IL-13 could induce a 2.9-fold increase in percentage of nuclear active YAP positive cells (nuclear-aYAP^+^/DAPI) (Figures 3A–C) while a 0.3-fold decrease in the ratio of ciliated cells (Figures 3A,D), albeit not statistically significant compared to untreated hNECs. The *in vitro* results were consistent with that of NIP Grade II and the trend between Grade II and Grade III NIP, suggesting the inhibitory effect of functional YAP on cilia in NIP. However, using IL-13 treatment as model, accompanied with nuclear-aYAP^+^/DAPI increasing, goblet cell ratio (MUC5AC^+^/DAPI) had a 2.9-fold increase (Figures 3B,E). The observation was not consistent with NIP tissue, potentially as IL-13 was unlikely the driver in NIP formation as compared to NP, or potentially due to the very low levels of MUC5AC^+^ cells in NIP suggesting that the higher levels of nuclear active YAP may have suppress differentiation of all epithelial cells. Nevertheless, we established that nuclear active YAP plays a role in reducing the differentiation of basal cells into ciliated cells during tissue remodeling processes.

### YAP Is Positively Correlated With Neutrophils in NIP Tissue

**FIGURE 4:**
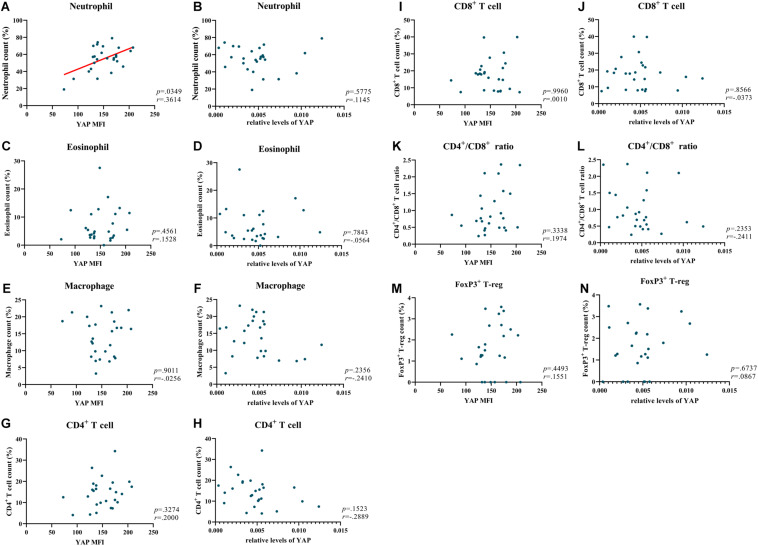
YAP is positively correlated with neutrophils in NIP. **(A,C,E,G,I,K,M)** Correlation analysis between MFI stained for total YAP and neutrophil count, eosinophil count, macrophage count, CD4^+^ T cell count, CD8^+^ T cell count, CD4^+^/CD8^+^ ratio, FoxP3^+^ T-reg count in NIP. **(B,D,F,H,J,L,N)** Correlation between YAP mRNA level and neutrophil count, eosinophil count, macrophage count, CD4^+^ T cell count, CD8^+^ T cell count, CD4^+^/CD8^+^ ratio, FoxP3^+^ T-reg count in NIP. YAP mRNA level was quantified by RT-qPCR assays and relative expression of the target gene was normalized to 2^− Δ^ ^CT^ with RPL13A. Correlation analysis was performed using the Spearman *r* test (*n* = 26).

In our previous study, we have explored the characteristics of inflammatory cells in NIP (Zhao L. et al., 2016). In the present study, we investigated the relationship between YAP expression and inflammatory cells. The detailed inflammatory cell data was described in Table 3. We explored all the relationship between inflammatory cells and YAP expression. Interestingly, even though no correlation between YAP mRNA level and neutrophils infiltration was observed (Figure 4B), we found that increased YAP protein is significantly correlated with increased neutrophils infiltration (Figure 4A), which implied that there was a connection between YAP and neutrophil infiltration. Neither YAP MFI nor YAP mRNA level had significant correlation with eosinophil count (Figures 4C,D), macrophage count (Figures 4E,F), CD4^+^ T cell count (Figures 4G,H), CD8^+^ T cell count (Figures 4I,J), CD4^+^/CD8^+^ ratio (Figures 4K,L), and FoxP3^+^ T-reg count (Figures 4M,N).

**TABLE 3:** Inflammatory or immune cell feature in NIP.

Inflammatory or immune cell count, median (1st, 3rd quartile)	
Eosinophil, %	4.85 (2.74, 11.11)
Neutrophil, %	57.14 (43.03, 69.56)
Macrophage, %	13.57 (8.24, 18.62)
CD4^+^T cell, %	14.49 (9.71, 18.29)
CD8^+^T cell, %	17.92 (8.52, 21.96)
CD4^+^/CD8^+^ ratio	0.77 (0.50, 1.36)
Regulator T cell (FoxP3^+^), %	1.28 (0.00, 2.50)

### NIP With Adverse Outcomes Has a High Level of YAP Expression

**FIGURE 5:**
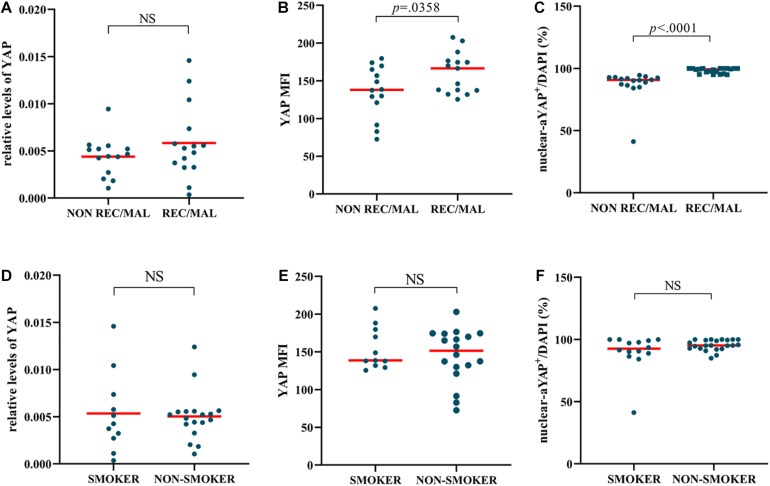
NIP with adverse outcomes has a higher level of YAP expression. **(A–C)** YAP mRNA level, Semi-quantitative analysis of mean fluorescence intensity (MFI) stained for total YAP, percentage of nuclear active YAP positive cells (nuclear-aYAP^+^/DAPI) in recurrence or malignancy (REC/MAL) status. **(D–F)** YAP mRNA level, YAP MFI, nuclear-aYAP^+^/DAPI in smoking status. The mRNA levels of YAP were quantified by RT-qPCR assays and relative expression of the target gene was normalized to 2^− Δ^ ^CT^ with RPL13A. Two group difference was analyzed with Mann–Whitney test. Red lines show median values. **(A,B)**, *n*(NON-REC/MAL) = 14, *n*(REC/MAL) = 15; **(C)**, *n*(NON-REC/MAL) = 16, *n*(REC/MAL) = 20; **(D,E)**, *n*(SMOKER) = 11, *n*(NON-SMOKER) = 18; **(F)**, *n*(SMOKER) = 14, *n*(NON-SMOKER) = 22.

When comparing YAP expression to NIP clinical data, we found that 15 had adverse outcomes, of which 15 (52%) had recurrence, and 3 (10%) underwent malignancy (Table 4). The results of immunofluorescence showed that the YAP MFI and percentage of nuclear YAP^+^ cells (nuclear-aYAP^+^/DAPI) in patients with recurrence or malignancy were both significantly higher than that in patients without adverse outcomes (Figures 5B,C). However, while YAP mRNA level was increased in patients with adverse outcomes, no statistical significance was observed between the two groups (Figure 5A). There was no difference in YAP expression with respect to smoking status (Figures 5D–F).

**TABLE 4:** Clinical characteristics of patients with NIP.

Clinical parameters	No. (%) (*n* = 29)
**Recurrent NIP**	
Yes	15 (52%)
No	14 (48%)
**Carcinogenesis**	
Yes	3 (10%)
No	26 (90%)

## Discussion

Our study has demonstrated that the expression of YAP, a core effector of the Hippo pathway, and proliferation level in NIP were higher than those of the NP group and control group. At the same time, we identified the positive relationship between proliferation level and YAP expression in NIP. In addition, YAP was also found to be related to epithelial differentiation and neutrophil infiltration in NIP. Clinically, there was also a correlation established between YAP and the adverse outcomes in NIP. Hence, further studies of YAP and the Hippo pathway in NIP may further explain its pathogenesis and may potentially serve as a target for treatment and prognostic marker for adverse outcomes.

The Hippo pathway, particularly its effector YAP, has been implicated in airway epithelial cell proliferation and differentiation (Zhao R. et al., 2014). In our previous study, we illustrated that YAP is involved in epithelial proliferation and remodeling in nasal polyps (Deng et al., 2019). In this study, we found that inverted papilloma similarly has increased YAP expression at levels higher than that of nasal polyp. YAP was found to be upregulated in NIP than that in healthy control and is positively correlated with the ratio of Ki-67 positive cells to basal cells. Because the ratio of Ki-67 positive cells to basal cells can represent cell proliferation, this result indicates that YAP was involved in the abnormal proliferation of NIP. Additionally, we also found that the severity of remodeling positively correlates with YAP expression. In our study, in both mRNA and protein levels, YAP expression was found to increase with grade, where Grade III levels were significantly higher than that of Grade II.

Yes-associated protein can also affect airway epithelium not only through promoting proliferation but also through affecting epithelial differentiation. In the mature lung airway of mice, the absence of YAP leads to an epithelial pattern of unlimited differentiation of airway epithelium. Conversely, the overexpression of YAP resulted in an opposite pattern of epithelial with a multilayer of poorly differentiated basal cells (Zhao R. et al., 2014). In our previous article, we demonstrated that YAP contributes to epithelial remodeling in NP (Deng et al., 2019). In this study, compared to Grade II, Grade III NIP has a decreased proportion of ciliated and goblet cells but an increased epithelial YAP expression. Therefore, YAP may contribute inverted papilloma remodeling in at least two different ways: promoting proliferation and suppressing normal differentiation. Interestingly, we also found that in Grade II NIP, the proportion of ciliated cells is negatively related to percentage of nuclear active YAP positive cells, although it is positively associated with YAP MFI. Conversely, the lack of correlation between YAP and goblet cells levels indicate that YAP is more likely to play a role in suppression of ciliated cells formation but not goblet cells, which may involve other pathways. This is evidenced by our *in vitro* hNECs study with IL-13 treatment, where the proportion of ciliated cells and percentage of nuclear active YAP positive cells show opposite trends, which is consistent with Grade II NIP and between Grade II and Grade III NIP. For goblet cells differentiation, no significant association of nuclear active YAP was found in Grade II NIP, as goblet cells levels are very low in NIP. However, in our recently published paper (Yuan et al., 2020), YAP may play a double role in repressing ciliated cell differentiation while promoting goblet cell differentiation, which was not seen in NIP. The difference in the levels of nuclear active YAP may lead to the discrepancies between NP and NIP observation, which remained to be further explored if there are other pathways influencing the goblet cell differentiation. These results may suggest that in lower grade NIP, YAP may not be the only factor influencing NIP differentiation. Additionally, the differentiation mechanism of NIP is also complex and has not been clarified. Further study is needed to explain the function of YAP on differentiation in NIP or other nasal diseases.

Finally, the proliferation level of NIP was not only higher than normal nasal epithelium but also than nasal polyps (Mumbuc et al., 2007; Meng et al., 2014). Our study confirmed this and indicated that the NIP epithelium was likely populated with cells with high proliferative ability. The increased proliferation level may explain why NIP has a faster growth rate and hence a higher clinical recurrence rate. In addition, we found that although there are many layers of basal cells in the NIP epithelium, they still exist near the side of the basement membrane. This is true even in areas where squamous metaplasia is very severe. These results can explain at least partly why NIP has the characteristic of growing into a matrix. The special location of the proliferative cells may partially explain the special structure of NIP: where cells in the apical side of NIP epithelia do not proliferate significantly compared to the basal side. Further study is needed to confirm this observation.

In our previous study, we also found a significant increase in neutrophils in Chinese NIP patients (Zhao L. et al., 2016). Interestingly, YAP was found to be related to neutrophils in this study. Although PCR that represents both epithelial and subcutaneous tissues did not show a correlation with neutrophils, YAP MFI that represents the epithelial levels of YAP showed a significant positive correlation between YAP and neutrophil count. This result is consistent with the high neutrophil level of NIP and is supported by literature (Kurz et al., 2018; Lv et al., 2018). Because one of the characteristics of NIP is the significant increase of neutrophils (Zhao L. et al., 2016), YAP’s regulation on neutrophils may at least partly contribute to the formation and development of NIP. This hypothesis would require further investigation. A small number of studies have shown that nicotine could up-regulate YAP expression in other tissue (Zhao Y. et al., 2014; Takahashi et al., 2020), but we did not detect difference of YAP expression between smoker and non-smoker in NIP. One reason may be that the influence of nicotine diluted by other factors affecting YAP; the other may be the difference of tissue. Further study is needed to explain the effect of nicotinic in nasal tissue.

The study, however, is not without its limitations. Firstly, some results were based on correlation analysis, and we did not further explore the mechanism of YAP function in NIP pathogenesis. Future experiments using gene-edited cell or animal models can be employed to investigate the mechanism in depth. Secondly, there were insufficient Grade I NIP samples for meaningful analysis of its association with YAP. In the future, more patients can be recruited to provide a more comprehensive elucidation of YAP expression in different grades of NIP. Finally, a part of our study, especially for those in tissues, only focused on total YAP expression, which includes both nuclear YAP and cytoplasmic YAP; only nuclear YAP is the functional form exerting its effect. This is due to the difficulty to clarify nuclear YAP in the tissue sections, and coupled with the lack of *in vivo* models, we were not able to further establish the mechanism between neutrophils infiltration and YAP in NIP. Further studies using *in vivo* animal models and YAP nuclear import blockers may be required for the investigation of YAP’s effect on neutrophils infiltration.

In this study, YAP was abnormally increased in NIP patients with adverse outcomes. At the same time, with the increase of the grade of NIP epithelial remodeling, the levels of YAP became higher. These results suggest that YAP may be an important metric of NIP and should be incorporated in future clinical assessment, including the severity of the disease and the normal airway function of the epithelium. Since the inhibitor of YAP, Verteporfin, has been used in the clinical treatment of other diseases (Bakri and Kaiser, 2004), YAP may be explored as a therapeutic target for NIP to reverse or alleviate the adverse outcomes of NIP.

## Data Availability Statement

The original contributions presented in the study are included in the article/Supplementary Material, further inquiries can be directed to the corresponding author/s.

## Ethics Statement

The studies involving human participants were reviewed and approved by the Institutional Review Boards of Qilu Hospital of Shandong University and 3rd Affiliated Hospital of Sun Yat-sen University. The patients/participants provided their written informed consent to participate in this study.

## Author Contributions

Q-TY, TY, X-MZ, RZ, and D-YW: study conception and design. PJ, Z-QH, LS, and JL: patient consent and enrollment. PJ, X-MZ, and X-XZ: surgery. TY, KST, HO, JL, S-ZZ, Q-MC, Q-WW, W-HW, and H-YD: acquisition of data or analysis and interpretation of data. W-FK, Y-YT, TL, H-JQ, X-KH, and Q-TY: quality control of the study. TY, Q-TY, and D-YW: drafting the article. All authors: involving in the study, revising the article critically for important intellectual content, and final approval of the version to be published.

## Conflict of Interest

The authors declare that the research was conducted in the absence of any commercial or financial relationships that could be construed as a potential conflict of interest. The handling editor declared a shared affiliation with several of the authors at the time of review.

## References

[B1] Atherton H. C. Jones G. Danahay H. (2003). IL-13-induced changes in the goblet cell density of human bronchial epithelial cell cultures: MAP kinase and phosphatidylinositol 3-kinase regulation. *Am. J. Physiol. Lung Cell. Mol. Physiol*. 285 L730–9. 12794003 10.1152/ajplung.00089.2003

[B2] Bakri S. J. Kaiser P. K. (2004). Verteporfin ocular photodynamic therapy. *Expert Opin. Pharmacother*. 5 195–203. 10.1517/14656566.5.1.195 14680447

[B3] Dellagrammaticas D. Lewis S. C. Gough M. J. Collaborators G. T. (2008). Is heparin reversal with protamine after carotid endarterectomy dangerous? *Eur. J. Vasc. Endovasc. Surg*. 36 41–44. 10.1016/j.ejvs.2008.01.021 18406179

[B4] Deng H. Sun Y. Wang W. Li M. Yuan T. Kong W. (2019). The hippo pathway effector yes-associated protein promotes epithelial proliferation and remodeling in chronic rhinosinusitis with nasal polyps. *Allergy* 74 731–742. 10.1111/all.13647 30362580

[B5] Katori H. Nozawa A. Tsukuda M. (2006). Histopathological parameters of recurrence and malignant transformation in sinonasal inverted papilloma. *Acta Otolaryngol*. 126 214–218. 10.1080/00016480500312554 16428203

[B6] Kim J. Jo H. Hong H. Kim M. H. Kim J. M. Lee J. K. (2015). Actin remodelling factors control ciliogenesis by regulating YAP/TAZ activity and vesicle trafficking. *Nat. Commun*. 6:6781. 10.1038/ncomms7781 25849865

[B7] Krouse J. H. (2000). Development of a staging system for inverted papilloma. *Laryngoscope* 110 965–968. 10.1097/00005537-200006000-00015 10852514

[B8] Kurz A. R. M. Catz S. D. Sperandio M. (2018). Noncanonical hippo signalling in the regulation of leukocyte function. *Trends Immunol*. 39 656–669. 10.1016/j.it.2018.05.003 29954663 PMC6528674

[B9] Lange A. W. Sridharan A. Xu Y. Stripp B. R. Perl A. K. Whitsett J. A. (2015). Hippo/Yap signaling controls epithelial progenitor cell proliferation and differentiation in the embryonic and adult lung. *J. Mol. Cell Biol*. 7 35–47. 10.1093/jmcb/mju046 25480985 PMC4400400

[B10] Li Y. Y. Li C. W. Chao S. S. Yu F. G. Yu X. M. Liu J. (2014). Impairment of cilia architecture and ciliogenesis in hyperplastic nasal epithelium from nasal polyps. *J. Allergy Clin. Immunol*. 134 1282–1292. 10.1016/j.jaci.2014.07.038 25201258

[B11] Liu J. Li Y. Y. Andiappan A. K. Yan Y. Tan K. S. Ong H. H. (2018). Role of IL-13Ralpha2 in modulating IL-13-induced MUC5AC and ciliary changes in healthy and CRSwNP mucosa. *Allergy* 73 1673–1685. 10.1111/all.13424 29405354

[B12] Lv Y. Kim K. Sheng Y. Cho J. Qian Z. Zhao Y. Y. (2018). YAP controls endothelial activation and vascular inflammation through TRAF6. *Circ. Res*. 123 43–56. 10.1161/circresaha.118.313143 29794022 PMC6014930

[B13] Mahoney J. E. Mori M. Szymaniak A. D. Varelas X. Cardoso W. V. (2014). The hippo pathway effector Yap controls patterning and differentiation of airway epithelial progenitors. *Dev. Cell* 30 137–150. 10.1016/j.devcel.2014.06.003 25043473 PMC6331061

[B14] Meng X. Wu X. Yuan Y. (2014). [Significances of COX-2, p21, Ki-67 expression and HPV infection in nasal inverted papilloma]. *Lin Chung Er Bi Yan Hou Tou Jing Wai Ke Za Zhi* 28 1823–1827. 25980147

[B15] Mumbuc S. Karakok M. Baglam T. Karatas E. Durucu C. Kibar Y. (2007). Immunohistochemical analysis of PCNA, Ki67 and p53 in nasal polyposis and sinonasal inverted papillomas. *J. Int. Med. Res*. 35 237–241. 10.1177/147323000703500208 17542411

[B16] Nielsen P. L. Buchwald C. Nielsen L. H. Tos M. (1991). Inverted papilloma of the nasal cavity: pathological aspects in a follow-up study. *Laryngoscope* 101 1094–1101. 1921638 10.1288/00005537-199110000-00012

[B17] Orlandi R. R. Rubin A. Terrell J. E. Anzai Y. Bugdaj M. Lanza D. C. (2002). Sinus inflammation associated with contralateral inverted papilloma. *Am. J. Rhinol*. 16 91–95. 10.1177/194589240201600204 12030363

[B18] Paz Silva M. Pinto J. M. Corey J. P. Mhoon E. E. Baroody F. M. Naclerio R. M. (2015). Diagnostic algorithm for unilateral sinus disease: a 15-year retrospective review. *Int. Forum Allergy Rhinol*. 5 590–596. 10.1002/alr.21526 25880633 PMC4830336

[B19] Roh H. J. Procop G. W. Batra P. S. Citardi M. J. Lanza D. C. (2004). Inflammation and the pathogenesis of inverted papilloma. *Am. J. Rhinol*. 18 65–74. 10.1177/194589240401800201 15152870

[B20] Sun Q. An L. Zheng J. Zhu D. (2017). Advances in recurrence and malignant transformation of sinonasal inverted papillomas. *Oncol. Lett*. 13 4585–4592. 10.3892/ol.2017.6089 28599459 PMC5453000

[B21] Takahashi T. Shiraishi A. Osawa M. (2020). Upregulated nicotinic ACh receptor signaling contributes to intestinal stem cell function through activation of Hippo and Notch signaling pathways. *Int. Immunopharmacol*. 88:106984. 10.1016/j.intimp.2020.106984 33182055

[B22] Yu F. X. Zhao B. Guan K. L. (2015). Hippo pathway in organ size control, tissue homeostasis, and cancer. *Cell* 163 811–828. 10.1016/j.cell.2015.10.044 26544935 PMC4638384

[B23] Yuan T. Zheng R. Liu J. Tan K. S. Huang Z. Q. Zhou X. M. (2020). Role of yes-associated protein in interleukin-13 induced nasal remodeling of chronic rhinosinusitis with nasal polyps. *Allergy* 76 600–604. 10.1111/all.14699 33301614

[B24] Zhao L. Li C. W. Jin P. Ng C. L. Lin Z. B. Li Y. Y. (2016). Histopathological features of sinonasal inverted papillomas in chinese patients. *Laryngoscope* 126 E141–7. 26434596 10.1002/lary.25694

[B25] Zhao R. Fallon T. R. Saladi S. V. Pardo-Saganta A. Villoria J. Mou H. (2014). Yap tunes airway epithelial size and architecture by regulating the identity, maintenance, and self-renewal of stem cells. *Dev. Cell* 30 151–165. 10.1016/j.devcel.2014.06.004 25043474 PMC4130488

[B26] Zhao R. W. Guo Z. Q. Zhang R. X. (2016). Human papillomavirus infection and the malignant transformation of sinonasal inverted papilloma: a meta-analysis. *J. Clin. Virol*. 79 36–43. 10.1016/j.jcv.2016.04.001 27085508

[B27] Zhao Y. Zhou W. Xue L. Zhang W. Zhan Q. (2014). Nicotine activates YAP1 through nAChRs mediated signaling in esophageal squamous cell cancer (ESCC). *PLoS One* 9:e90836. 10.1371/journal.pone.0090836 24621512 PMC3951250

