# Supplementary material for: Abnormal Expression of YAP Is Associated With Proliferation, Differentiation, Neutrophil Infiltration, and Adverse Outcome in Patients With Nasal Inverted Papilloma
Source: Front Cell Dev Biol. 2021 Apr 15;9:625251. doi: 10.3389/fcell.2021.625251 (PMC8083899; doi:10.3389/fcell.2021.625251)
Supplement: Supplementary file 1 [file Data_Sheet_1.docx]

**Supplementary materials**

**Abnormal expression of YAP is associated with proliferation, differentiation, neutrophil infiltration, and adverse outcome in patients with nasal inverted papilloma**

Tian Yuan^1,5#^, Rui Zheng^1#^, Xiang-min Zhou^2#^, Peng Jin^2^, Zhi-qun Huang^3^, Xiao-xue Zi^2^, Qing-wu Wu^1^, Wei-hao Wang^1^, Hui-yi Deng^1^, Wei-feng Kong^1^, Hui-jun Qiu^1^, Sui-zi Zhou^4^, Qian-min Chen^4^, Yan-yi Tu^2^, Tao Li^2^, Jing Liu^5,6^, Kai-Sen Tan^5,6,7,8^, Hsiao Hui Ong^5,6^, Li Shi^2^, Zhuang-gui Chen^9^, Xue-kun Huang^1^, Qin-tai Yang^1*^, De-yun Wang^5,6*^

| **Table S1. Clinical Characteristics of Active YAP Staining Samples** | | | |
| --- | --- | --- | --- |
| **Clinical Parameters** | **Control(n=10)**  **No. (%)** | **NP(n=18)**  **No. (%)** | **NIP(n=36)**  **No. (%)** |
| **Age, y, median (1st & 3rd interquartile)** | 40 (30,51) | 41 (37,53) | 49 (38,52) |
| **Gender** |  |  |  |
| **Male** | 7(70%) | 12(67%) | 28(78%) |
| **Female** | 3(30%) | 6(33%) | 8(22%) |
| **Smoking** |  |  |  |
| **Smoker** | 1(20%) | 4(22%) | 14(39%) |
| **Nonsmoker** | 9(80%) | 14(78%) | 22(61%) |
| **Epithelial remodeling grading** |  |  |  |
| **Grade I** | - | - | 1(3%) |
| **Grade II** | - | - | 16 (44%) |
| **Grade III** | - | - | 19(53%) |
| NP = Nasal polyps; NIP = Nasal inverted papilloma. | | | |


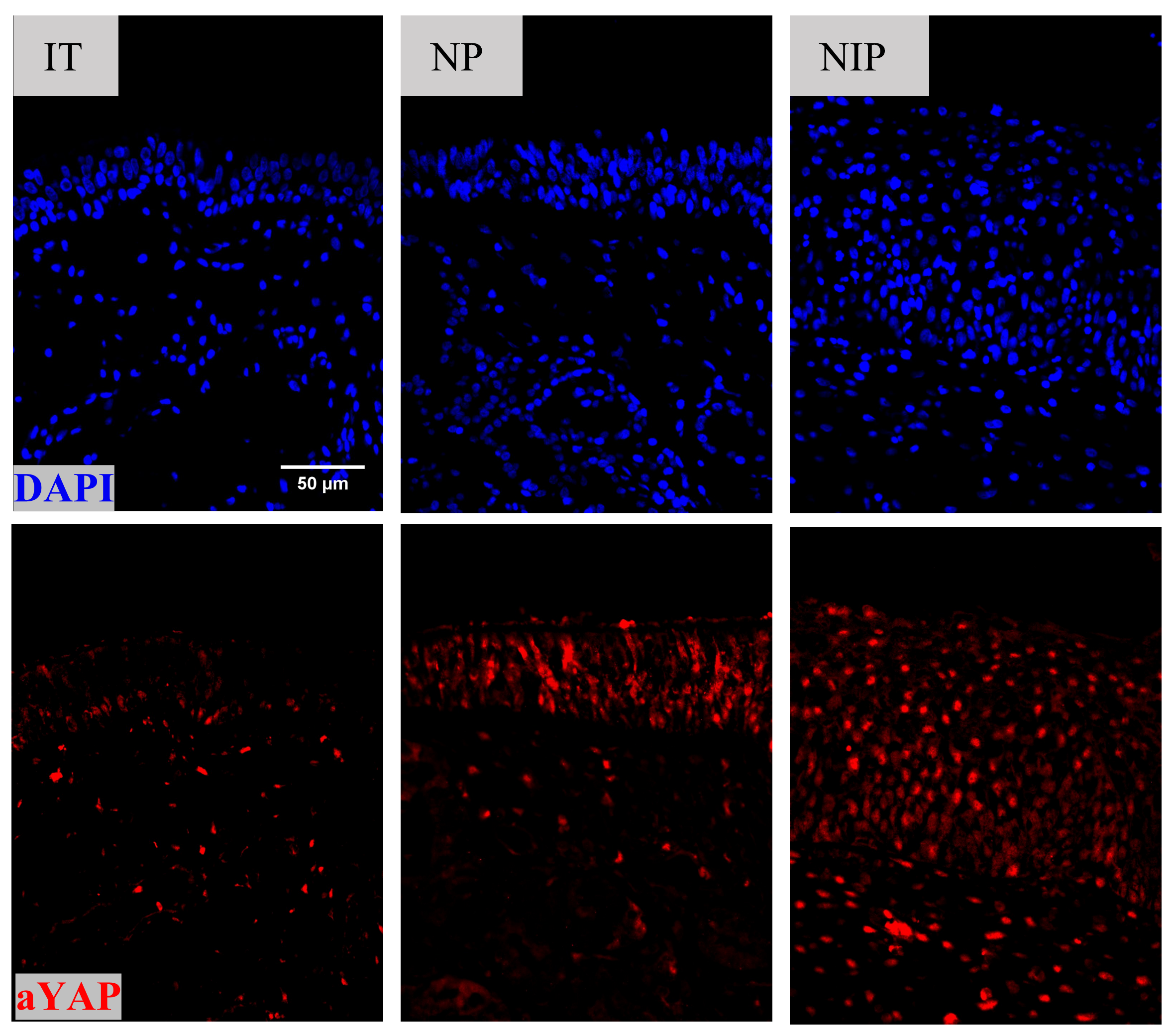


**Fig. S1. Active YAP (aYAP) and DAPI IF staining in control IT, NP, and NIP tissues. Single channel,** **×400 magnification, scale bar=50μm.**


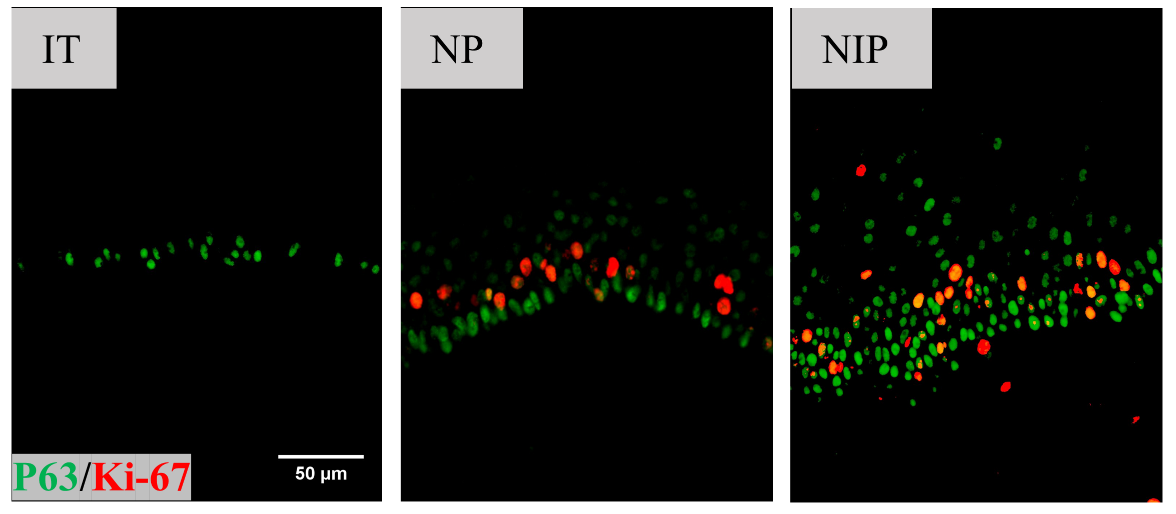


**Fig. S2. Ki-67 and P63 double IF staining without DAPI in control IT, NP, and NIP tissues. ×400 magnification, scale bar=50μm.**


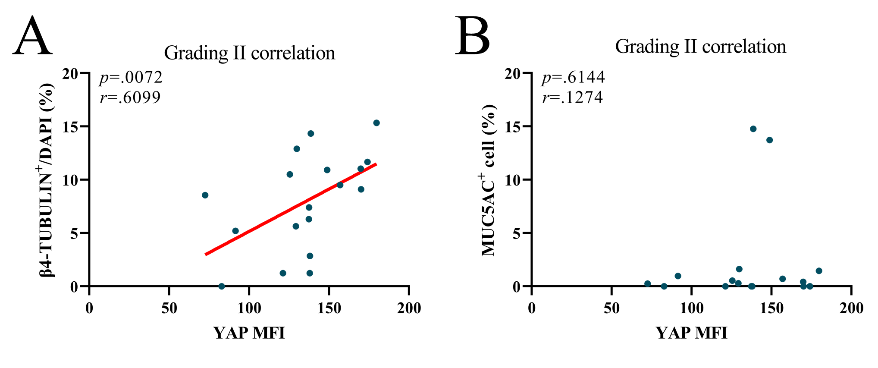


**Fig. S3. Correlation analysis between semi-quantitative analysis of mean fluorescence intensity (MFI) stained for total YAP and ciliated cell ratio (β4-TUBULIN^+^/DAPI), goblet cell ratio (MUC5AC^+^/DAPI) in Grade II NIP tissues. Correlation analysis was performed using the Spearman r test. n=18.**
